# Combined Effects of Microplastics and Biochar on the Removal of Polycyclic Aromatic Hydrocarbons and Phthalate Esters and Its Potential Microbial Ecological Mechanism

**DOI:** 10.3389/fmicb.2021.647766

**Published:** 2021-04-30

**Authors:** Xinwei Ren, Jingchun Tang, Lan Wang, Hongwen Sun

**Affiliations:** ^1^Key Laboratory of Pollution Processes and Environmental Criteria (Ministry of Education), Tianjin Engineering Center of Environmental Diagnosis and Contamination Remediation, College of Environmental Science and Engineering, Nankai University, Tianjin, China; ^2^School of Agriculture and Biology, Shanghai Jiao Tong University, Shanghai, China; ^3^Shanghai Yangtze River Delta Eco-Environmental Change and Management Observation and Research Station, Ministry of Science and Technology, Ministry of Education, Shanghai, China; ^4^Shanghai Urban Forest Ecosystem Research Station, National Forestry and Grassland Administration, Shanghai, China

**Keywords:** microplastics, biochar, PAHs, PAEs, microbes, removal

## Abstract

Microplastics (MPs) have been attracting wide attention. Biochar (BC) application could improve the soil quality in the contaminated soil. Currently, most studies focused on the effect of MPs or BC on the soil properties and microbial community, while they neglected the combined effects. This study investigated the combined effects of BC or ball-milled BC (BM) and polyethylene plastic fragments (PEPFs) and degradable plastic fragments (DPFs) on the removal of polycyclic aromatic hydrocarbons (PAHs) and phthalate esters (PAEs) from the PAH-contaminated soil and the potential microbial ecological mechanisms. The results showed that BC or BM combined with PEPF could accelerate the removal of PAHs and PAEs. PEPF combined with BM had the most significant effect on the removal of PAHs. Our results indicating two potential possible reasons contribute to increasing the removal of organic pollutants: (1) the high sorption rate on the PEPF and BC and (2) the increased PAH-degrader or PAE-degrader abundance for the removal of organic pollutants.

## Introduction

Plastic is used in a wide range of products (Plastics Europe, 2018/2019) due to its low cost, light weight, and durability (Cozar et al., 2014). The extensive use of plastics resulted in the release of plastic waste into the environment at an alarming rate (Geyer et al., 2017). Larger plastics break into the smaller pieces, resulting in the formation of microplastics (MPs) (Barnes et al., 2009; Rillig, 2012). The concept of MPs (<5 mm) was proposed by Thompson (2004), characterized by small size, large amount, wide distribution (Rillig, 2012), and the adsorption characteristics (Law and Thompson, 2014). MPs are prone to absorb the pollutants on its surface (Besseling et al., 2013; Hodson et al., 2017) or accumulate the specific microbes such as pathogens (Foulon et al., 2016), thus posing potential risks to the ecosystem through the food web and food chain (Browne et al., 2013; Wright et al., 2013).

One of the main MP sources in soil is plastic film. Previous studies showed that plastic could reach 40–60% of the surface of home gardens soil (Huerta Lwanga et al., 2016). The total amount MPs of farmland in North America and the plastic content on the surface soil of the solid waste enrichment region in Germany could reach 44,000–300,000 and 43,454 t^⋅^a^–1^ (Ng et al., 2018), respectively. The degradable plastic fragments (DPF) has drawn wide concern recently due to the possibility of replacing traditional plastic film, although its effects on ecosystem in the short- or long-term are still not fully understood (Bandopadhyay et al., 2018). A previous study showed that both polyethylene (PE) MPs and DPF-MPs could affect soil carbon and nitrogen content (Qi et al., 2020). The additives used in the plastic production process such as phthalate esters (PAEs) could also pose a serious problem to the soil ecosystem after being released into the soil (Wang et al., 2013). Kong et al. (2012) showed that vegetable soil had the highest PAE value, indicating that PAEs in plastic film could enter into the soil. In addition, MPs are high-molecular polymers with characteristics such as hydrophobic properties and high octanol–water partition coefficient (Kow), thus making it easy for them to adsorb hydrophobic organic compounds (HOCs) (Rillig, 2012; Horton et al., 2017). MPs could accumulate microbes such as Actinobacteria, Bacteroidetes, and Proteobacteria, which could degrade the polymer (Zhang et al., 2019), polycyclic aromatic hydrocarbons (PAHs), and dioxins (Gatheru Waigi et al., 2017). MPs could also affect some microorganisms involved in the biogeochemical cycle, such as Burkholderiaceae, Xanthobacteraceae, and Pseudomonadaceae (Kielak et al., 2016), and could raise the abundance of Bradyrhizobiaceae and Burkholderiaceae, which associated with nitrogen fixation (Fei et al., 2020) and changed the soil nutrient condition. Therefore, study on the effects of MPs on the structure and function of soil microbial community could help us deeply and comprehensively understand the fate of contaminants in the environment and the potential mechanism.

Biochar (BC) could improve the soil quality and enhance the adsorption and degradation of pollutants to reduce the microbial toxicity of the pollutants, so it is commonly used for the improvement of organic contaminated soil, although the effects of BC on degradation of organic pollutants showed mixed results currently (He et al., 2018). Some studies showed that BC could accelerate the degradation rate of organic pollutants as a result of enhancing the microbial activity or the microbial utilization of the organic pollutants on the BC. BC has direct or indirect effects on microorganisms (Zhu et al., 2017). BC could directly provide protection and nutrient to the soil microorganisms (Jia et al., 2017; Luo et al., 2018) or provide the microbes a more suitable living environment by changing the soil properties. In addition, BC could release dissolved organic carbon (DOC) into soil solution after application and could directly alter the properties of soil DOC (Dittmar et al., 2012). The DOC of BC is labile and more susceptible to biodegradation than BC; therefore, it plays an important role in controlling microbial activities (Wang B. et al., 2017; Bao et al., 2020). A recent study showed that soil DOC could be the main factor influencing the total relative abundances of dominant PAH-degraders (Ding et al., 2020), while the results of the other studies were opposite. During the preparation process of BC, PAHs could also be produced. Lyu et al. (2016) showed that BC produced at 300–400°C had higher PAHs or PCDD/DF toxicity than BC prepared at a temperature higher than 400°C. The mineral elements, volatile organic compounds (VOCs), and free radicals contained in BC can affect microbial activity and change the soil microbial community structure (Abel et al., 2013) and soil enzyme activity, which could further affect soil function. Therefore, the environmental and ecological effects caused by BC have drawn great concern (Wang C. et al., 2017).

Biochar is widely used as soil amendment; however, MP fragments exist in the soil at the same time, which could change the structure and function of the soil and microbial community (Ding et al., 2017; Rillig, 2018). The present study focused on the combined effects of MP fragments and BC on the soil physical and chemical properties, and the degradation of pollutants in PAH-polluted soil. We aimed to illustrate (1) the response of soil DOM and its components to the combined effects of plastic fragments and BC; (2) the influence of plastic fragments and BC on the removal of PAHs and PAEs; and (3) the potential microbial ecological mechanisms.

## Materials and Methods

### Microplastics and Biochar

The PE plastic bags were purchased from a subsidiary agricultural product store in Tianjin, China. The degradable mulching film was obtained from an agricultural product store, composed of polylactic acid (PLA) and polybutylene adipate-*co*-terephthalate (PBAT). The film was cut into small pieces by using sharp blades and scissors of ∼4.5 mm in length. Before use, the fragments were sterilized under ultraviolet light for 20 min to minimize the potential microbial contamination. The BC used in this study was made from poplar woodchips. Before use, the collected woodchips were washed, dried, and pass through a 100 mesh sieve (Xiao et al., 2020). Then the woodchips were heated at 500°C in a muffle furnace for 3 h under an oxygen-limited condition, cooled to room temperature, washed with distilled water, and dried at 80°C for 24 h. The yield of the BC was about 30.85%. The obtained BC was divided into two parts: one part was stored in the dark before use and labeled as BC, and the other one was used to prepare the ball-milled BC (BM) (Lyu et al., 2018; Xiao et al., 2020). The BC was placed in an agate jar (500 ml). The ratio of BC to agate ball was 1:50 (BC-to-ball mass ratios). The agate jar was sealed and placed into a planetary ball mill machine (F-P2000, Focucy, Hunan, China). The ball milling was performed at a speed of 200 rpm for 24 h at room temperature; and the rotation direction was changed every 6 h. The resulting BC was labeled as BM.

The contents of C, H, and N of the BC were determined by an element analyzer (vario EL CUBE, Germany). The contents of C, H, and N were 75.74, 5.12, and 0.78% for BC and 71.79, 3.88, and 0.84% for BM. The morphologies of BC and BM were characterized by a field-emission scanning electron microscope (SEM; JSM-7800F, Rigaku, Tokyo, Japan). The specific surface areas, pore volume, and pore size of BC and BM were determined by Micromeritics ASAP2460 (Atlanta, United States) based on the N_2_ adsorption–desorption method. The data were analyzed according to the methods of Brunauer–Emmett–Tuller (BET) and Barrett–Joyner–Halenda (BJH). The Fourier-transform infrared (FT-IR) spectroscopy was used to analyze the surface functional groups on the surfaces of BC and BM by TENSOR 37 (Bruker, Germany) in the region of 400–4,000 cm^–1^ with a resolution of 4 cm^–1^.

### Experimental Design

The PAH-contaminated soil was brought back to the laboratory, air-dried, sieved through 2-mm mesh, and then thoroughly mixed for the subsequent microcosm experiment. The initial concentrations (Supplementary Table 1) and the structural formulas (Supplementary Figure 1) of 16 priority PAHs are listed in the Supplementary Material. Beakers measuring 350 ml were used in this study. Before use, the beakers were washed and sterilized. For each beaker, 300-g soil was mixed with the BC and MPs, resulting in nine different treatments (Supplementary Table 2). BC, BM, D, and P represented BC, BM, degradable plastic fragments (DPFs), and PE plastic fragments (PEPFs). After water was added [equivalent to the 60% of the field capacity (w/w)], the samples were incubated at 22°C (80% relative humidity). Each treatment was carried out in three replicates. Samples from each pot were collected on the 105th day of the experiment. The samples were separated into two parts: one part was stored in the refrigerator for testing soil physical and chemical properties, and the other part was stored at −80°C for molecular microbiological analysis.

### Analysis of Soil Properties

Soil DOM solution was extracted by adding deionized water (ratio of soil:water was 1:5; 8-g soil with 40 ml of deionized water) in a 50-ml centrifuge tube according to the method described in the previous studies (Jaffrain et al., 2007; Ren et al., 2020). Soil extracts were centrifuged at 3,500 rpm for 15 min and then filtered by pre-rinsed 0.45-μm cellulose-acetate membranes (Solarbio, Beijing, China). The filtered solutions were analyzed by multi N/C 3100 (Analytik Jena AG, Germany) for DOM analysis. The functional groups of the subsamples were measured by UV-Vis spectrophotometer (LAMBDA-35, PerkinElmer, United States). UV-Vis absorption from 200 to 500 nm (1-nm steps) was measured in a 10-mm quartz cuvette with deionized water as blank. The specific UV absorbance (SUVA) at 210, 250, 254, 260, 265, 272, 280, 285, 300, 340, 350, 365, 400, 436, and 465 nm was measured for all samples. Detailed information as well as the wavelengths used in this study and their corresponding organic functional groups is shown in Supplementary Table 3. The soil NO_3_^–^–N was determined by UV spectrophotometry (TU-1810DASPC, PERSEE, Beijing, China) according to the method GB/T 32737-2016; the extracts were tested at 220 and 275 nm.

### Analysis of Polycyclic Aromatic Hydrocarbons and Phthalate Esters

Quantification of 16 PAHs and six priority PAEs [di(2-ethylhexyl)phthalate (DEHP), dibutyl phthalate (DBP), dimethyl phthalate (DMP), diethyl phthalate (DEP), butyl phenyl phthalate (BBP), and di-*n*-octyl phthalate (DNOP)] in soil was carried out by gas chromatography–mass spectrometry (GC-MS; GC7890B/MS5977B) according to the method of US EPA 8270E-2018. Briefly, a 10-g soil sample was used for each sample and then placed into the extraction pool. After diatomite was added and acetone/hexane (1:1, v/v) was stirred and extracted according to accelerated solvent extraction (ASE) method, the sample was concentrated to 1 ml via rotary evaporation method. Then a 200-μl sample was put into the GC vial, and the internal standard was added and mixed evenly. The internal standard substances and their corresponding contents are listed in the Supplementary Table 4. Then analysis was carried out by GC-MS with DB-5MS capillary column at a flow of 1.5 ml.min^–1^. The samples were injected at 280°C without splitting injection. The GC oven temperature was held at 45°C for 3 min, then increased by 30°C.min^–1^ from 45°C to 280°C, and then followed by 10°C .min^–1^ from 280°C to 300°C; and then the temperature was held at 300°C for 4.5 min. The temperature of the ion source was set as 230°C.

### DNA Extraction, PCR, and 16S rRNA Sequencing

Microbial community genomic DNA was extracted from soil samples using the FastDNA^®^, Spin Kit (MP Biomedicals, Santa Ana, CA, United States) according to manufacturer’s instructions. The extracted DNA was checked on 1% agarose gel electrophoresis, and the concentration and purity were determined using NanoDrop 2000 UV-Vis spectrophotometer (Thermo Scientific, Wilmington, United States). The primers containing barcode and adaptor used in this study are listed in Supplementary Table 5. The triplicate PCR products were recovered by using the AxyPrep DNA Gel Extraction Kit (Axygen Biosciences, Union City, CA, United States) following the manufacturer’s instructions and quantified using Quantus^TM^ Fluorometer (Promega, United States). Purified amplicons were pooled in equimolar; and paired-end sequencing (2 × 250) was performed on an Illumina MiSeq platform (Illumina, San Diego, CA, United States) according to the standard protocols by Majorbio Bio-Pharm Technology Co. Ltd. (Shanghai, China). The detailed PCR method and bioinformatic analysis are listed in the Supplementary Material (the PCR mixtures and amplification conditions).

### Statistical Analysis

Statistical analyses were performed using IBM SPSS Statistics 24.0. One-way ANOVA was used to determine the effect of different treatments on PAHs, PAEs, and soil DOM. The means of significant effects at *p* < 0.05 were then compared using the Duncan multiple-range test. Each treatment was conducted in three replicates. The error bars in the figures were expressed as the standard deviation (SD); and all of the data in the figures are presented as means ± SD. Operational taxonomic unit (OTU)-level alpha diversity indices, Chao1 (Chao, 1984), abundance-based coverage estimators (ACEs) (Chao and Yang, 1993), and Shannon index (Shannon, 1948) were calculated using the OTU table in Mothur. Figures were visualized by R 3.6.1 (R Core Team, 2019) and RStudio 1.1.463 (RStudio Team, 2018). Package information and detailed data analysis method were listed in our previous study (Ren et al., 2020).

## Results and Discussion

### Characterization of Biochar

The SEM results of the original BC and BM are shown in Supplementary Figure 2. The surface of BC was rough and porous. The porous size was at the micron scale (Supplementary Figure 2A,C) and common in pyrolytic materials (Zhou et al., 2013). After ball milling, the BC became fine particles (diameter < 1 μm) (Supplementary Figures S2B,D), indicating that ball milling could effectively transform the BC into ultrafine particles.

Supplementary Table 6 shows the BET results. The specific surface area of the BC could be increased from 85.7 to 321.9 m^2^.g^–1^ after ball milling. The pore volume and average pore size of the BM were higher than those of BC. Previous studies have shown that ball milling technology could not only increase the external and internal surface area by reducing the size of the BC but could also increase the internal surface area by getting through the inner pore network.

The FT-IR spectra of the BC and BM are shown in Supplementary Figure 3. Before analysis, all samples were dried and kept dry to avoid the interference of moisture. Vibration peaks were observed at 3,443 cm^–1^ (–OH) and 1,385 cm^–1^ (O-C–O). After ball milling, the extension of BC shifted from 1,610 to 1,612 cm^–1^ at C-O. In addition, the peak appeared at 1,171 cm^–1^, indicating that the C–O functional groups increased after ball milling.

### Effects of Different Treatments on Dissolved Organic Matter and Relative Functional Group Characteristics in Soil

**FIGURE 1:**
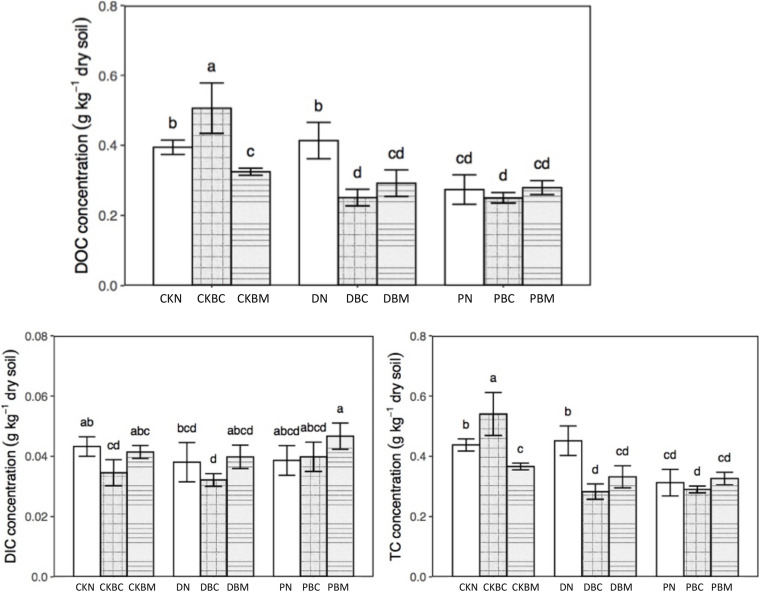
DOC, DIC, and TC concentration of different treatments. Different letters mean significant differences, and the same letters mean no significant difference, Duncan (*p* < 0.05). DOC, dissolved organic carbon; DIC, dissolved inorganic carbon; TC, total dissolved carbon.

**FIGURE 2:**
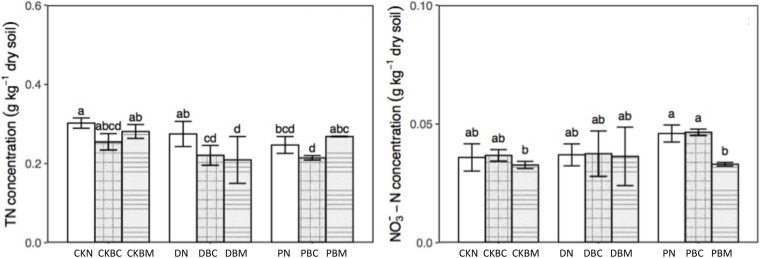
TN and NO_3_^–^–N concentration of different treatments.

The DOC and total dissolved carbon (TC) had the same changing tendency (Figure 1). In soil without plastic fragments, the content of DOC and TC in CKBC (1% biochar + no adding plastics fragments) was significantly higher than that of CKN (no adding biochar + no adding plastics fragments) (*p* = 0.001; *p* = 0.002, LSD, *p* < 0.05), while in CKBM (1% ball-milled biochar + no adding plastics fragments), it was significantly lower than that of CKN (*p* = 0.036; *p* = 0.030, Least-Significant Difference (LSD), *p* < 0.05). The potential reason might be that BC could increase the soil DOC (Cui, 2021) with the higher adsorption capacity of ball milling BC (Kumar et al., 2020) due to the larger specific surface area after ball milling (Xiang et al., 2020). In soil with plastic fragments, both BC and BM decreased the DOC and TC. DN slightly increased the soil DOC compared with CKN, which might be due to its degradable characteristics, while PN decreased the soil DOC, which might be because the soil environment could change the surface of PEPF and increase the surface functional groups, resulting in the sorption of DOC on PEPF. A recent study showed that the co-existence of PE-MPs and BC could increase the sorption of ion (Li et al., 2021). A previous study showed that the soil DOC with rice straw and PLA-MPs was significantly lower than that in soil with rice straw alone when rice straw was used as the carbon source (Chen et al., 2020). Our result showed that the co-existence of BC and plastic fragments could significantly decrease the soil DOC compared with the soil with BC alone, which was similar to the previous results. As for the dissolved inorganic carbon (DIC), CKBC and DBC decreased its contents. The changing trends of total dissolved nitrogen (TN) and nitrate nitrogen (NO_3_^–^–N) are shown in Figure 2. DBC (1% biochar + degradable plastic fragments) and DBM (1% ball-milled biochar + degradable plastic fragments) significantly decreased the content of TN (*p* = 0.024, *p* = 0.008, LSD, *p* < 0.05), while they had no significant effect on NO_3_^–^–N compared with DN; PBM (1% ball-milled biochar + PE plastics fragments) significantly decreased the content of NO_3_^–^–N compared with PN (*p* = 0.016, LSD, *p* < 0.05).

**FIGURE 3:**
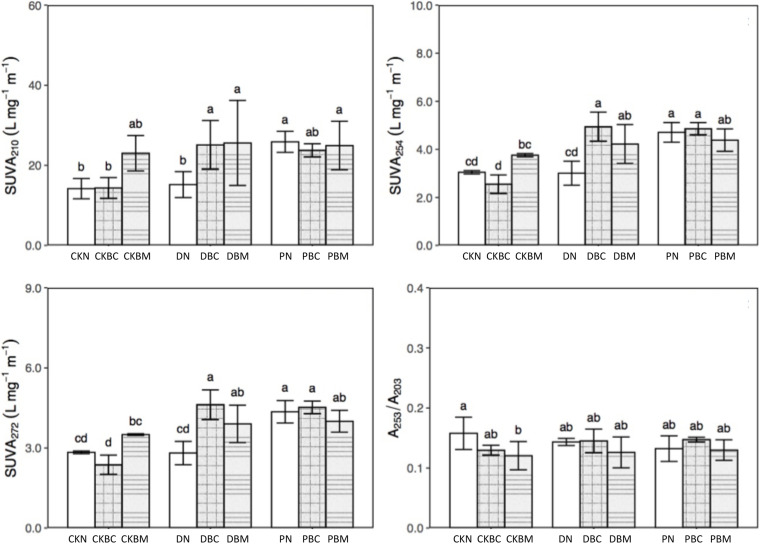
Specific ultraviolet absorbance (SUVA) at different wavelengths: SUVA_210_, SUVA_254_, SUVA_272_, and A_253_/A_203_ of different treatments. SUVA_210_ means amines, SUVA_254_ and SUVA_272_ mean aromatic substances, and A_253_/A_203_ represent aromatic ring substituents.

Figure 3 and Supplementary Table 7 show the results of the SUVA at different wavelengths. PN significantly increased the content of amines (SUVA_210_) and aromatic substances (SUVA_254_, SUVA_260_, SUVA_272_, SUVA_280_, and SUVA_285_) compared with CKN, while it had no significant effect on the DOC molecular weight (A_250_/A_365_), aromatic ring substituents (A_253_/A_203_), the ratio of C-C and C-O (A_265_/A_465_), and the soil aggregation degree (A_300_/A_400_). DN had no significant influence on characteristic functional groups. DBC could change the components of DOC and significantly raise the contents of amines and aromatic substances as compared with CKBC, which might be because the degradable MPs could change the components of DOC (Chen et al., 2020).

Therefore, DBC significantly decreased DOC and increased the amines and aromatic substances, indicating that the combined effects could change the components of DOC. CKBM or DBM significantly increased the content of amines and aromatic substances as compared with CKN or DN, respectively. Compared with CKN, DN had no significant effect, while DBM could significantly decrease the DOC and increase the amines and aromatic substances, indicating that the combined effects could change the components of DOC (Figures 1, 3). PN could decrease the DOC as well as the contents of amines and aromatic substances, illustrating that PN could alter the components of DOC. PBC (1% biochar + PE plastics fragments) or PBM had similar results with PN. Therefore, the combined effect of DPF and BC could change the components of DOC, while the potential reason for PN changing the DOC contents was due to its own characteristics.

### Effects of Microplastics on the Degradation of Phthalate Esters and Polycyclic Aromatic Hydrocarbons

The changes of PAHs and PAEs in groups CKN, CKBC, CKBM, DBC, DBM, PBC, and PBM are shown in Tables 1, 2. The degradation rates of PAHs and PAEs are shown in Tables 3, 4, respectively. In this study, PAHs of CKBC and CKBM were higher than those in CKN. The contents of total PAHs of PBC and PBM were lower than those of the other groups. Therefore, PN combined with BC potentially accelerated the removal of total PAHs. As for the degradation rate, CKN had the highest degradation rate of PAHs (2 + 3) (Table 4). For PAHs (4), PBC had the highest degradation rate 61.75 ± 6.65%, followed by PBM 56.03 ± 8.32%, both of which had a significant difference with CKN (*p* = 0.002, *p* = 0.007, LSD, *p* < 0.05). The degradation rate of PAHs (5 + 6) in PBC and PBM could reach 77.10 ± 4.02% and 80.18 ± 3.87%, respectively. The total PAH degradation rates of PBC and PBM were 49.74 ± 8.43% and 45.09 ± 9.98%, which were higher than those of other treatments. Among the six priority controlled PAEs, DEHP and DBP were detected (Table 2). PAEs as the common pollutants in soil have a negative effect on soil enzyme and microbes and are often regarded as the “environmental endocrine-disrupting chemicals” (Xu et al., 2020). Our results showed that the contents of PAEs in CKBC and CKBM were significantly higher than those in CK. The contents of PAEs in CKBM were higher than CKBC. PAEs had a lower content in PBC and PBM than other groups. As for the degradation rate (Table 4), DBP, DEHP, and total PAE degradation rates in group PBC and PBM were significantly higher than those in the other groups.

**TABLE 1:** The concentration of 16 PAHs in soil under the different treatments (mg^⋅^kg^–1^).

	**CKN**	**CKBC**	**CKBM**	**DBC**	**DBM**	**PBC**	**PBM**
Naphthalene (Nap)	5.30 ± 0.78^b^	5.40 ± 0.20^b^	5.73 ± 0.67^b^	7.57 ± 0.50^a^	6.27 ± 1.21^b^	3.73 ± 0.75^c^	3.97 ± 0.84^c^
Acenaphthylene (AcPy)	0.33 ± 0.06^c^	0.43 ± 0.06^c^	2.30 ± 0.10^a^	0.43 ± 0.06^c^	1.97 ± 0.35^a^	0.27 ± 0.12^c^	1.30 ± 0.44^b^
Acenaphthene (Acp)	7.47 ± 1.61^e^	12.53 ± 0.68^de^	19.20 ± 0.26^ab^	18.80 ± 2.49^ab^	17.93 ± 2.74^bc^	12.23 ± 3.90^de^	13.13 ± 4.28^cd^
Fluorene (Flu)	4.23 ± 0.67^f^	8.33 ± 0.35^cd^	7.20 ± 0.53^de^	10.73 ± 0.50^bc^	6.57 ± 1.00^def^	6.27 ± 1.95^def^	4.73 ± 1.50^ef^
Phenanthrene (Phe)	6.33 ± 0.46^d^	8.13 ± 0.40^cd^	12.30 ± 1.41^a^	8.83 ± 0.76^c^	9.27 ± 1.50^bc^	3.73 ± 1.02^e^	5.90 ± 2.14^d^
Anthracene (Ant)	0.20 ± 0.00^abc^	0.20 ± 0.00^abc^	0.27 ± 0.12^ab^	0.23 ± 0.06^abc^	0.17 ± 0.06^bc^	0.13 ± 0.06^c^	0.13 ± 0.06^c^
Fluoranthene (FL)	2.90 ± 0.10^b^	3.13 ± 0.15^ab^	3.67 ± 0.64^ab^	3.13 ± 0.32^ab^	2.90 ± 0.46^b^	1.57 ± 0.46^c^	1.90 ± 0.62^c^
Pyrene (Pyr)	2.20 ± 0.00^b^	2.40 ± 0.10^b^	2.77 ± 0.47^ab^	2.53 ± 0.23^ab^	2.17 ± 0.31^b^	1.20 ± 0.35^c^	1.43 ± 0.47^c^
Benzo[*a*]anthracene (BaA)	1.47 ± 0.06^abc^	1.63 ± 0.06^ab^	1.63 ± 0.32^ab^	1.53 ± 0.15^abc^	1.30 ± 0.20^bc^	0.77 ± 0.21^d^	0.73 ± 0.31^d^
Chrysene (Chr)	1.37 ± 0.06^bc^	1.67 ± 0.15^abc^	1.70 ± 0.36^abc^	1.80 ± 0.20^ab^	1.47 ± 0.25^bc^	0.70 ± 0.26^d^	0.80 ± 0.20^d^
Benzo[*b*]fluoranthene (BbF)	1.90 ± 0.10^ab^	1.90 ± 0.10^ab^	1.83 ± 0.25^ab^	1.93 ± 0.25^ab^	1.50 ± 0.26^b^	0.87 ± 0.31^c^	0.90 ± 0.36^c^
Benzo[*k*]fluorathene (BkF)	0.87 ± 0.21^abc^	0.90 ± 0.10^abc^	0.83 ± 0.25^bc^	1.20 ± 0.20^a^	0.87 ± 0.25^abc^	0.33 ± 0.06^de^	0.23 ± 0.06^e^
Benzo[*a*,*h*]anthracene (BaP)	1.28 ± 0.14^abc^	1.44 ± 0.18^ab^	1.36 ± 0.45^ab^	1.32 ± 0.18^ab^	1.04 ± 0.22^bc^	0.36 ± 0.13^d^	0.28 ± 0.09^d^
Indeno[1,2,3-*cd*]pyrene (InP)	1.77 ± 0.06^a^	1.87 ± 0.15^a^	1.37 ± 0.21^bc^	1.63 ± 0.25^ab^	1.07 ± 0.15^cd^	0.47 ± 0.12^ef^	0.40 ± 0.10^f^
Dibenzo[*a*,*h*]anthracene (DbA)	0.48 ± 0.02^ab^	0.52 ± 0.04^a^	0.42 ± 0.07^b^	0.45 ± 0.06^ab^	0.30 ± 0.05^c^	0.13 ± 0.04^d^	0.12 ± 0.03^d^
Benzo[*g*,*h*,*i*]perylene (BghiP)	1.97 ± 0.06^a^	2.10 ± 0.20^a^	1.47 ± 0.21^bc^	1.80 ± 0.30^ab^	1.13 ± 0.21^cd^	0.50 ± 0.17^ef^	0.37 ± 0.15^f^
Total PAHs	40.06 ± 2.57	52.59 ± 2.04	64.05 ± 5.52	63.94 ± 6.04	55.91 ± 9.01	33.26 ± 9.67	36.33 ± 11.43

*Different letters after the same column of numbers indicate that there was significant difference between different treatment groups under the same particle size (*p* < 0.05), the same as in the succeeding tables. PAH, polycyclic aromatic hydrocarbon.*

**TABLE 2:** The concentration of PAEs in soil under the different treatments (mg^⋅^kg -1).

	**DEHP**	**DBP**	**Total PAEs**
CKN	0.70 ± 0.10^abc^	0.40 ± 0.17^a^	1.10
CKBC	0.73 ± 0.06^abc^	0.40 ± 0.00^a^	1.13
CKBM	0.80 ± 0.00^a^	0.43 ± 0.06^a^	1.23
DBC	0.73 ± 0.06^abc^	0.43 ± 0.06^a^	1.17
DBM	0.67 ± 0.06^abc^	0.43 ± 0.06^a^	1.10
PBC	0.47 ± 0.15^d^	0.23 ± 0.06^b^	0.70
PBM	0.57 ± 0.15^cd^	0.30 ± 0.00^ab^	0.87
Initial concentrations	0.83 ± 0.06^a^	0.40 ± 0.00^a^	1.23

*PAE, phthalate ester; DEHP, di(2-ethylhexyl)phthalate; DBP, dibutyl phthalate; PAEs, phthalate esters. Different letters mean significant differences, and the same letters mean no significant difference.*

**TABLE 3:** The degradation rate of 16 PAHs in soil under the different treatments (%).

	**PAHs (2 + 3)**	**PAHs (4)**	**PAHs (5 + 6)**	**Total PAHs**
CKN	45.13 ± 3.89^a^	28.31 ± 0.80^b^	28.81 ± 1.16^c^	39.46 ± 2.24^ab^
CKBC	19.46 ± 2.16^bc^	20.18 ± 1.98^b^	24.85 ± 3.44^c^	20.53 ± 1.78^bc^
CKBM	−8.05 ± 3.27^d^	11.74 ± 9.28^b^	37.26 ± 7.04^bc^	3.21 ± 4.82^c^
DBC	−7.13 ± 5.52^d^	18.68 ± 4.55^b^	28.15 ± 6.15^c^	3.38 ± 5.27^c^
DBM	3.07 ± 8.91^cd^	29.21 ± 6.27^b^	49.07 ± 5.64^b^	15.50 ± 7.86^c^
PBC	39.39 ± 10.13^ab^	61.75 ± 6.65^a^	77.10 ± 4.02^a^	49.74 ± 8.43^a^
PBM	32.95 ± 12.15^ab^	56.03 ± 8.32^a^	80.18 ± 3.87^a^	45.09 ± 9.98^a^

*The number in the parentheses represents the rings of polycyclic aromatic hydrocarbons (PAHs). The degradation rate = (*C*_0_ - *C*_*t*_)/*C*_0_ × 100%, where *C*_0_ is the initial PAH contents of different classifications (2 + 3 rings, 4 rings, 5 + 6 rings, and total PAHs; listed in Supplementary Table 4), and *C*_*t*_ is the final PAH contents of different classifications on day 105 (listed in Table 1). Different letters mean significant differences, and the same letters mean no significant difference.*

**TABLE 4:** The degradation rate of PAEs in soil under the different treatments (%).

	**DBP**	**DEHP**	**Total PAEs**
CKN	0.00 ± 25.00^b^	15.66 ± 6.96^bc^	10.57 ± 12.42^bc^
CKBC	0.00 ± 0.00^b^	11.64 ± 4.02^bc^	7.86 ± 2.71^bc^
CKBM	−8.33 ± 8.33^b^	3.61 ± 0.00^c^	-0.27 ± 2.71^c^
DBC	−8.33 ± 8.33^b^	11.64 ± 4.02^bc^	5.15 ± 2.71^c^
DBM	−8.33 ± 8.33^b^	19.68 ± 4.02^bc^	10.57 ± 0.00^bc^
PBC	41.67 ± 8.33^a^	43.78 ± 10.63^a^	43.09 ± 9.39^a^
PBM	25.00 ± 0.00^ab^	31.73 ± 10.63^ab^	29.54 ± 7.17^ab^

*The degradation rate = (*C*_0_ - *C*_*t*_)/*C*_0_ × 100%, where *C*_0_ is the initial concentrations of different PAEs (DBP, DEHP, and total PAEs; listed in Table 2), and *C*_*t*_ is the final PAE concentrations of different PAEs on day 105 (listed in Table 2). PAEs, phthalate esters; DBP, dibutyl phthalate; DEHP, di(2-ethylhexyl)phthalate. Different letters mean significant differences, and the same letters mean no significant difference.*

A previous study suggested that the effects of BC on degradation of pollutants in soil showed mixed results (He et al., 2018): accelerating or reducing the degradation rate. There are several reasons for reducing the natural degradation rate by BC. Firstly, the pyrolysis process could introduce PAHs to BC, and the content of PAHs is correlated with the producing temperature, conditions, and type of raw materials (Keiluweit et al., 2012; Wang C. et al., 2017). Our results showed that the contents of PAHs in CKBC and CKBM were higher than those in CKN, which might be due to the PAHs produced in BC production process entering into the soil with BC (Table 1). Another potential reason was the adsorption characteristics of BC increasing the adsorption and immobilization of organic pollutants in soil resulting in the declined degradation. The strong sorption of organic pollutants on the carbonaceous materials could reduce their microbial degradation leading to high residues of the pollutants in the soil (Ding et al., 2020). Some studies also illustrated that BC showed the potential toxicity to microbes induced by the organic compounds on the BC (Zhu et al., 2017; Yaashikaa et al., 2020). These might be the potential possibilities for the PAHs and PAEs in CKBC and CKBM higher than those in CKN. In addition, the contents of PAHs and PAEs in CKBM were higher than those in CKBC. This might be due to its larger specific surface area resulting in more adsorption sites. The combined effects of PE-MPs and BC on the significant decrease of PAHs and PAEs in soil might be due to the sorption effect of PE-MPs on the organic pollutants (Jiménez-Skrzypek et al., 2021) or the selective effects on the specific microbes.

### Effects of Microplastics on the α-Diversity of Microbes

**FIGURE 4:**
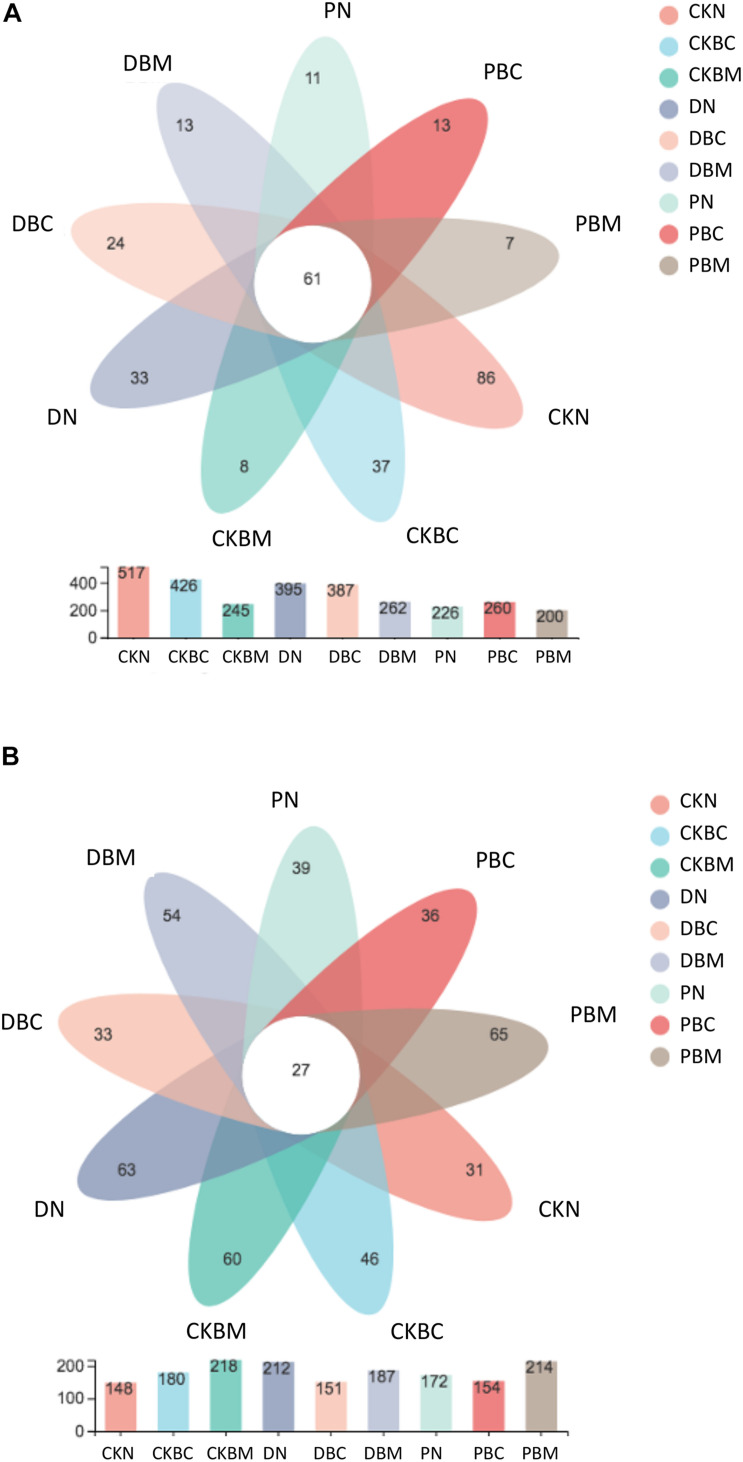
Venn diagram of operational taxonomic unit (OTUs) of **(A)** bacteria and **(B)**.

The microbial α-diversity indices and the Venn diagram of common and specific OTUs are shown in Figure 4 and Supplementary Table 8. The bacterial and fungal OTUs ranged from 200 to 517 and from 148 to 214, respectively. The common OTUs of bacterial and fungal community were 61 and 27, respectively. For bacteria, CKN had the highest specific OTU number (86), and the PBM had the lowest number (7). For fungi, CKN had the lowest number (31), and PBM had the highest number (65) (Figure 4). In soil without plastic fragments, BC decreased the bacterial OTUs (CK < CKBC < CKBM), while it increased the fungal OTUs (CK > CKBC > CKBM). Compared with CKN, the combined effects of plastic fragments and BC could decrease the OTUs of bacteria, while it increased those of fungi. A previous study showed that MPs could decrease the microbial α-diversity (Zhang et al., 2019). In our study, the result of bacterial community was consistent with the existing research results. In soil without BC, DN and PN decreased the Shannon and Chao indices of bacteria compared with CKN. Compared with CKN and CKBM, CKBC had the lowest values of bacterial diversity index, although CKBM had the higher diversity than CKBC, while it had a lower richness index. DBM and PBM increased the bacterial community diversity, while they decreased the richness. As for the fungi, DBM increased the diversity, while it decreased the richness; PBM decreased the diversity, while it increased the richness. The influences of BC on diversity and richness of the microbial community were affected by the types of MPs. CKBC and CKBM increased the richness of fungal community and CKBM increased its diversity index. Both DBC and PBC decreased the diversity and richness of fungi. DBM decreased the diversity and richness, and PBM decreased the diversity, while it increased the richness.

### Effects of Microplastics on the Microbial Community Structure

**FIGURE 5:**
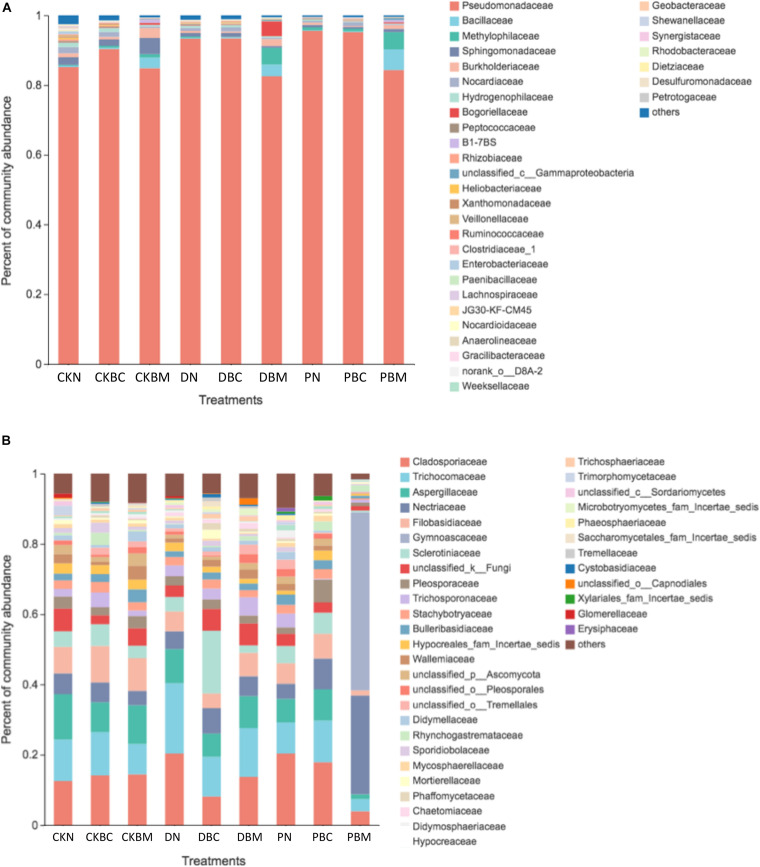
Community composition at the family level: **(A)** bacteria and **(B)** fungi.

The microbial community structure at the family level is shown in Figure 5. The results showed that Pseudomonadaceae was the dominant bacteria in all the treatments. Compared with CKN, plastic fragments increased the abundance of Pseudomonadaceae, from 85.15% in CKN to 93.28% (DN) and 95.51% (PN), and decreased the abundance of Sphingomonadaceae, from 2.18% in CKN to 0.48% (DN) and 0.92% (PN). In soil with plastic fragments, BC had no significant influence on the abundance of Pseudomonadaceae, while BM decreased the abundance of Pseudomonadaceae and increased the abundance of Methylophilaceae, Bacillaceae, Burkholderiaceae, and Bogoriellaceae. The abundance of Pseudomonadaceae decreased from 93.28% in DN to 82.53% in DBM; the abundance of Methylophilaceae, Bacillaceae, Burkholderiaceae, and Bogoriellaceae increased from 0.34 to 4.84%, 0.29 to 3.35%, 0.60 to 2.02%, and 0.12 to 4.23% in DN and DBM. The abundance of Pseudomonadaceae decreased from 95.51% (PN) to 84.28% (PBM); the abundance of Methylophilaceae, Bacillaceae, Burkholderiaceae, and Bogoriellaceae increased from 0.90 to 4.93%, 0.16 to 5.96%, 0.63 to 1.10%, and 0.01 to 0.38% in PN and PBM. In soil with plastic fragments, BC had no significant influence on the abundance of the microbes. BM decreased the abundance of Pseudomonadaceae, while it increased Methylophilaceae, Bacillaceae, Burkholderiaceae, and Bogoriellaceae. The influence of DPF and PEPF was similar. Methylophilaceae played an important role in the removal of PAHs at the later stage (Lu et al., 2019). Some species in the family Burkholderiaceae have degradable genes (Oyehan and Al-Thukair, 2017).

As for the fungi, previous study showed that *Fusarium* had the assistant effect on the removal of PAHs (Rafin et al., 2009). *Acremonium*, *Pleurotus ostreatus*, *Trichoderma*, *Trametes versicolor*, and *Pleurotus* are associated with the removal of PAHs (Liu et al., 2017). PAHs can be immobilized by the particularity of fungal extracellular enzymes (Baldantoni et al., 2017). A previous study also showed that the content of PAHs decreased after adding fungi (Bellino et al., 2019). Therefore, the study of fungal community structure could help to further understand the mechanism of PAH degradation. The main fungal community at the family level included Cladosporiaceae, Trichocomaceae, Aspergillaceae, Nectriaceae, and Filobasidiaceae. Except for DBC, DBM, and PBM, the dominant family in other treatments was Cladosporiaceae. In addition, several researches showed that *Fusarium* in the family Hypocreaceae had a degradable effect on DPB (Wang et al., 2016; Cheng et al., 2018).

Our study showed that adding BC or ball milling BC to PAH-contaminated soil with plastic residual could reduce the content of PAHs in soil and accelerate its removal. PBM had the highest removal rate. The reason for the highest removal rate might be due to that combined effects that BM and plastic fragments had on the selective effect on the specific microbes associated with PAHs, thus accelerating the removal of PAHs. BM had a similar influence on the microbial structure in soil with DPF and PEPF, decreased the abundance of Pseudomonadaceae, and increased the abundance of Methylophilaceae, Bacillaceae, Burkholderiaceae, and Bogoriellaceae. These microbes are all related to PAHs and could act as the indicator microbes related to PAH degradation. Some fungal communities are also associated with PAHs (Potin et al., 2004; Aranda et al., 2017; Vasudevan et al., 2018). The change of fungal community might lead to the changes in bacterial degradation capacity (Wu et al., 2020). In PBM, the abundance of Nectriacea increased. A previous study showed that some species in Nectriaceae played a key role in the removal of PAHs (Lladó et al., 2013; Brusetti et al., 2018), which might be one of the reasons for the decrease of PAH content in the treatment of PBM. Therefore, except for the sorption effect of MPs and BC on the organic pollutants potentially accelerating the removal rate, the selective enrichment effect of the plastic debris and BC on the specific bacterial community potentially helps to accelerate the removal of PAHs.

## Conclusion

In this study, the combined effects of plastic fragments and BC on the soil properties, the removal of pollutants, and the microbial community were tested. Our results illustrated that the combined effects of PEPF with BC or BM could accelerate the removal of PAHs. The influence of DPF on soil DOC was related to the soil carbon content. BM decreased the soil DOC, which might be due to its strong adsorption while irrelevant with the types of plastics. The combined effect of DPF with BC or BM could change the component of DOC, while the change of DOC caused by PEPF was associated with its own characteristics. Our results indicating two potential possible reasons contribute to increasing the removal of organic pollutants, (1) the high sorption rate on the PEPF and BC and (2) the increased PAH-degrader or PAE-degrader abundance for the removal of organic pollutants.

## Data Availability Statement

The original contributions presented in the study are included in the article/Supplementary Material, further inquiries can be directed to the corresponding author/s.

## Author Contributions

XR did the experiment and prepared the manuscript. JT designed the experiment and prepared the manuscript. LW did the data treatment analysis work. HS prepared the manuscript. All the authors contributed to the article and approved the submitted version.

## Conflict of Interest

The authors declare that the research was conducted in the absence of any commercial or financial relationships that could be construed as a potential conflict of interest.
