# Supplementary material for: Combined Effects of Microplastics and Biochar on the Removal of Polycyclic Aromatic Hydrocarbons and Phthalate Esters and Its Potential Microbial Ecological Mechanism
Source: Front Microbiol. 2021 Apr 30;12:647766. doi: 10.3389/fmicb.2021.647766 (PMC8120302; doi:10.3389/fmicb.2021.647766)
Supplement: Supplementary file 1 [file Data_Sheet_1.docx]

**Supplementary material**

**Combined Effects of Microplastics and Biochar on the Removal of Polycyclic Aromatic Hydrocarbons and Phthalate Esters and Its Potential Microbial Ecological Mechanism.**

Xinwei Ren^1,2,3,4^, Jingchun Tang^1^*, Lan Wang^1^*, Hongwen Sun^1^

1. Key Laboratory of Pollution Processes and Environmental Criteria (Ministry of Education), Tianjin Engineering Center of Environmental Diagnosis and Contamination Remediation, College of Environmental Science and Engineering, Nankai University, Tianjin 300350, China
2. School of Agriculture and Biology, Shanghai Jiao Tong University, 800 Dongchuan Rd., Shanghai, 200240, China
3. Shanghai Yangtze River Delta Eco-Environmental Change and Management Observation and Research Station, Ministry of Education, Ministry of Science and Technology, 800 Dongchuan Rd, Shanghai, 200240, China
4. Shanghai Urban Forest Ecosystem Research Station, National Forestry and Grassland Administration, 800 Dongchuan Rd., Shanghai, 200240, China

***Correspondence:**

Jingchun Tang

E-mail: tangjch@nankai.edu.cn;

Lan Wang

E-mail: envwangl@nankai.edu.cn

**Contents**

**The PCR mixtures and amplification conditions**

**Bioinformatic analysis**

**Tables**

Table S1 The initial concentrations of PAHs in contaminated soil.

Table S2 Treatments with their corresponding group names.

Table S3Key wavelengths selected in this study and their corresponding reference works.

Table S4 The internal standard substances and their corresponding contents.

Table S5 Primers used in PCR amplification.

Table S6 Pore structure parameters of biochar and ball-milled biochar.

Table S7 Specific ultraviolet absorbance (SUVA) at different wavelengths.

Table S8 The soil microbial α-diversity indexes in different treatments.

**Figures**

Figure S1 Structural Formula of 16 PAHs.

Figure S2SEM for biochar and ball-milling biochar: A, C-biochar; B, D-milling biochar.

Figure S3 FT-IR spectra for biochar/ ball-milling biochar: BC-biochar, BM-ball-milled biochar.

**References**

**The PCR mixturesand amplification conditions**

(1) The total volume of PCR reaction was 20 µL, listed as follow:

Components of the PCR reaction system

| bacteria/fungi | Adding volume(µL) |
| --- | --- |
| 5 × FastPfuBuffer/10× Buffer | 4/2 |
| 2.5mMdNTPs | 2 |
| BSA | 0.2 |
| ForwardPrimer (5µM) | 0.8 |
| ForwardPrimer (5µM) | 0.8 |
| FastPfuPolymerase/rTaq Polymerase | 0.4/0.2 |
| TemplateDNA | 10 ng |
| ddH2O | Adding to 20 |

(2) The bacterial 16S rRNAV3-V4and fungi ITS1 regions were amplified by an ABI GeneAmp® 9700 PCR thermocycler (ABI, CA, USA). The PCR amplification was performed as follows:

i95°C，3min；

iiBacteria:27 cycles $\times$(95°C,15s;annealing at 55°C,30s;extension at 72°C,45s);

Fungi:35 cycles$\times$(95°C,15s;annealing at 55°C,30s;extension at 72°C,45s);

iiisingle extensionat 72°C,10min.

**Bioinformatic analysis**

The raw gene sequencing reads were demultiplexed, quality-filtered by QIIME (v.1.9.1) and merged by FLASH according to the following criteria:

i raw sequencing reads were truncated at any site receiving an average quality score of <20 over a 50 bp sliding window, and the truncated reads < 50 bp were discarded, reads containing ambiguous characters were also discarded;

iionly overlapping sequences > 10 bp were assembled according to their overlapped sequence. The maximum mismatch ratio of overlap region is 0.2. Reads that could not be assembled were discarded;

iiiSamples were distinguished according to the barcode and primers, and the sequence direction was adjusted, exact barcode matching, 2 nucleotide mismatches in primer matching.

Operational taxonomic units (OTUs) with 97% similarity cutoff were clustered using UPARSE (v.7.0.1090, http://drive5.com/uparse/), and chimeric sequences were identified and removed. The taxonomy of each OTU representative sequence was analyzed by RDP Classifier (v.2.11, https://sourceforge.net/projects/rdp-classifier/) against the database Silva for bacteria and UNITE for fungi using confidence threshold of 0.7. PICRUSt2 was used to predict the functional potential of bacterial communities based on the normalizing bacterial OTUs table according to Kyoto Encyclopedia of Genes and Genomes (KEGG) database at level 2.

**Table S1The initialconcentrations of PAHs in contaminated soil**

Table S1 Theinitial concentrations of PAHs in contaminated soil.

| PAHs | Molecular formula | Molecular weight | concentrations  (mg$\cdot$kg^-1^) |
| --- | --- | --- | --- |
| Naphthalene (Nap) | C_10_H_8_ | 128.17 | 6.20±0.26 |
| Acenaphthylene (AcPy) | C_12_H_8_ | 152.19 | 0.50±0.00 |
| Acenaphthene (Acp) | C_12_H_10_ | 154.21 | 13.07±0.35 |
| Fluorene (Flu) | C_13_H_10_ | 166.22 | 10.97±0.15 |
| Phenanthrene (Phe) | C_14_H_10_ | 178.23 | 12.57±1.37 |
| Anthracene (Ant) | C_14_H_10_ | 178.23 | 0.20±0.00 |
| Fluoranthene (FL) | C_16_H_10_ | 202.25 | 4.27±0.25 |
| Pyrene (Pyr) | C_16_H_10_ | 202.25 | 3.17±0.12 |
| Benzo[a]anthracene (BaA) | C_18_H_12_ | 228.29 | 1.63±0.15 |
| Chrysene (Chr) | C_18_H_12_ | 228.29 | 2.00±0.17 |
| Benzo[b]fluoranthene (BbF) | C_22_H_12_ | 252.30 | 2.87±0.21 |
| Benzo[k]fluorathene (BkF) | C_22_H_12_ | 252.30 | 1.27±0.32 |
| Benzo [a, h] anthracene (BaP) | C_20_H_12_ | 252.32 | 1.61±0.18 |
| Indeno[1,2,3-cd] pyrene (InP) | C_22_H_12_ | 276.33 | 2.43±0.23 |
| Dibenzo [a, h] anthracene (DbA) | C_22_H_14_ | 278.35 | 0.63±0.06 |
| Benzo [g, h, i] perylene (BghiP) | C_22_H_12_ | 276.33 | 2.80±0.26 |
| Total PAHs |  |  | 66.17±1.81 |

**Table S2Treatments with their corresponding group names**

Table S2 Treatments with their corresponding group names

| Group setting | N | 1% BC | 1% BM |
| --- | --- | --- | --- |
| CK | CKN | CKBC | CKBM |
| 1% D | DN | DBC | DBM |
| 1% P | PN | PBC | PBM |

Notes:

N represented the no adding biochar (BC) or ball-milled biochar (BM)

CK represented no adding degradable plastic fragments (D) or PE plastics fragments (P)

**Table S3Key wavelengths selected in this study and their corresponding reference works**

The unit absorbance values (SUVA) of 210, 254, 260, 265, 272, 280, 285, 300, 340, 350, 400, 436 and 465were calculated as the ratio of absorbance value to DOC content, and then referred to as SUVA_210_, SUVA_254_, SUVA_260_, SUVA_272_, SUVA_280_,SUVA_285_, SUVA_340_, A_250_/A_365_, A_253_/A_203_,A_265_/A_465_,A_300_/A_400_.

Table S3 Key wavelengths selected in this study and their corresponding reference works

| **Wavelength (nm)** | **Properties** | **Reference** |
| --- | --- | --- |
| 210 | amine substance  molecular structure dominated by -CO-NH-functional groups | (Li et al., 2017) |
| 254 | aromaticity | (Croué et al., 2003; Hur and Schlautman, 2003; Jaffrain et al., 2007) |
| 260 | hydrophobic C content | (Dilling and Kaiser, 2002; Jaffrain et al., 2007) |
| 272 | aromaticity | (Jaffrain et al., 2007) |
| 280 | hydrophobic C content, humification index, apparent molecular size | (Jaffrain et al., 2007; Kalbitz et al., 2003) |
| 285 | humification index | (Kalbitz et al., 2000) |
| 340 | color | (Scott et al., 2001) |
| A_250_/A_365_ | reflects the molecular status of DOC | (Li et al., 2017; Li et al., 2014) |
| A_253_/A_203_ | relative abundance of functional groups | (Chen et al., 2002) |
| A_265_/A_465_ | relative abundance of functional groups | (Chen et al., 2002) |
| A_300_/A_400_ | characterization of humic substances and the molecular status of DOC | (Jaffrain et al., 2007) |

**Table S4The internal standard substances and their corresponding contents**

Table S4The internal standard substances and their corresponding contents

|  | Internal standard substances | Contents |
| --- | --- | --- |
| PAHs | Naphthalene | 5 μg$\cdot$ml |
|  | Acenaphthylene | 5 μg$\cdot$ml |
|  | Acenaphthene | 5 μg$\cdot$ml |
|  | Fluorene | 5 μg$\cdot$ml |
| PAEs | 1,4-Dichlorobenzene-d4 | 5 μg$\cdot$ml |
|  | Naphthalene-d8 | 5 μg$\cdot$ml |
|  | Acenaphthene-d10 | 5 μg$\cdot$ml |
|  | Phenanthrene-d10 | 5 μg$\cdot$ml |
|  | Chrysene-d12 | 5 μg$\cdot$ml |
|  | Perylene-d12 | 5 μg$\cdot$ml |

**Table S5 Primers used in PCR amplification**

Table S5 Primers used in PCR amplification

|  | Primers | Sequences | Amplification length |
| --- | --- | --- | --- |
| Bacterial | 338F | 5’-ACTCCTACGGGAGGCAGCA-3’ | 468 bp |
|  | 806R | 5’-GGACTACHVGGGTWTCTAAT-3’ |  |
| Fungi | ITS1F | 5’- CTTGGTCATTTAGAGGAAGTAA-3’ | 300 bp |
|  | ITS2R | 5’- GCTGCGTTCTTCATCGATGC -3’ |  |

**Table S6Pore structure parameters of biochar and ball-milled biochar**

Table S6Pore structure parameters of biochar and ball-milled biochar

| Parameters of pore structure | Unit | Biochar | Ball-milled biochar |
| --- | --- | --- | --- |
| BET surface area | m^2^⋅g^-1^ | 85.6851 | 321.9100 |
| BJH adsorption surface area (1.7000 nm～300.0000 nm) | m^2^⋅g^-1^ | 23.745 | 60.780 |
| BJH desorption surface area (1.7000 nm ~ 300.0000 nm) | m^2^⋅g^-1^ | 5.7470 | 36.1457 |
| Single point adsorption pore volume (<283.8936 nm) | cm^3^⋅g^-1^ | 0.047224 | 0.208214 |
| BJH adsorption pore volume (1.7000 nm ~ 300.0000 nm) | cm^3^⋅g^-1^ | 0.018972 | 0.111473 |
| BJH desorption pore volume (1.7000 nm ~ 300.0000 nm) | cm^3^⋅g^-1^ | 0.010156 | 0.085600 |
| BET adsorption average pore diameter | nm | 2.20452 | 2.58723 |
| BJH adsorption average pore diameter | nm | 3.1960 | 7.3362 |
| BJH desorption average pore diameter | nm | 7.0689 | 9.4728 |

**Table S7Specific ultraviolet absorbance (SUVA) at different wavelengths**

Table S7Specific ultraviolet absorbance (SUVA) at different wavelengths: SUVA_260_, SUVA_280_, SUVA_285_,SUVA_340_, A_250_/A_365_, A_265_/A_465_ and A_300_/A_400_of different treatments.

|  | SUVA_260_ | SUVA_280_ | SUVA_285_ | SUVA_340_ | A_250_/A_365_ | A_265_/A_465_ | A_300_/A_400_ |
| --- | --- | --- | --- | --- | --- | --- | --- |
| **CKN** | 2.9913±0.0596cd | 2.6668±0.0570cd | 2.5762±0.0572cd | 1.8040±0.0911cd | 2.1964±0.0874 a | 2.3070±0.1401 a | 1.6545±0.0941 a |
| **CKBC** | 2.5140±0.3851d | 2.2335±0.3288d | 2.1517±0.3202d | 1.4597±0.2062d | 2.2284±0.0569 a | 2.4289±0.0909 a | 1.7432±0.1377 a |
| **CKBM** | 3.6560±0.0513bc | 3.2640±0.0383bc | 3.1458±0.0436bc | 2.1963±0.0611bc | 2.2260±0.1098 a | 2.3045±0.0492 a | 1.7172±0.1001 a |
| **DN** | 2.9434±0.4804cd | 2.6310±0.4168cd | 2.5338±0.4050cd | 1.7594±0.2557cd | 2.1740±0.0686 a | 2.3774±0.0669 a | 1.7591±0.0403 a |
| **DBC** | 4.8592±0.5631a | 4.3585±0.5224a | 4.1862±0.4816a | 2.9173±0.2785a | 2.1860±0.1181 a | 2.4160±0.0453 a | 1.7489±0.0961 a |
| **DBM** | 4.1030±0.7515ab | 3.6510±0.6517ab | 3.5209±0.6252ab | 2.4594±0.3794ab | 2.1898±0.1323 a | 2.3789±0.0564 a | 1.6898±0.0380 a |
| **PN** | 4.5791±0.4485a | 4.0778±0.3848a | 3.9192±0.3832a | 2.6953±0.3739a | 2.3066±0.0898 a | 2.3875±0.1022 a | 1.7626±0.1190 a |
| **PBC** | 4.7716±0.2543a | 4.2909±0.2025a | 4.1089±0.2036a | 2.8729±0.1273a | 2.1529±0.0248 a | 2.3330±0.0429 a | 1.7118±0.0311 a |
| **PBM** | 4.2433±0.4377ab | 3.7644±0.3560ab | 3.6373±0.3414ab | 2.5418±0.1908ab | 2.1985±0.2833 a | 2.3070±0.1401 a | 1.6545±0.0941 a |

**Table S8The soil microbial α-diversity indexes in different treatments**

Table S8The soil microbial α-diversity indexes in different treatments

|  | **Treatment** | **CKN** | **CKBC** | **CKBM** | **DN** | **DBC** | **DBM** | **PN** | **PBC** | **PBM** |
| --- | --- | --- | --- | --- | --- | --- | --- | --- | --- | --- |
| Bacteria | Shannon | 1.14 | 0.76 | 0.87 | 0.57 | 0.55 | 0.93 | 0.36 | 0.39 | 0.84 |
|  | Simpson | 0.715 | 0.808 | 0.718 | 0.865 | 0.866 | 0.683 | 0.909 | 0.901 | 0.71 |
|  | Chao | 667 | 609 | 500 | 606 | 575 | 422 | 397 | 431 | 387 |
|  | Coverage | 0.995 | 0.995 | 0.996 | 0.995 | 0.995 | 0.996 | 0.997 | 0.996 | 0.997 |
| Fungi | Shannon | 3.92 | 3.92 | 4.22 | 3.84 | 3.91 | 4.04 | 4.08 | 3.77 | 1.88 |
|  | Simpson | 0.043 | 0.045 | 0.038 | 0.067 | 0.038 | 0.04 | 0.038 | 0.05 | 0.335 |
|  | Chao | 150 | 186 | 221 | 215 | 157 | 189 | 200 | 162 | 229 |
|  | Coverage | 1.000 | 1.000 | 1.000 | 1.000 | 1.000 | 1.000 | 1.000 | 1.000 | 1.000 |

**Figure S1 Structural Formula of 16 PAHs**


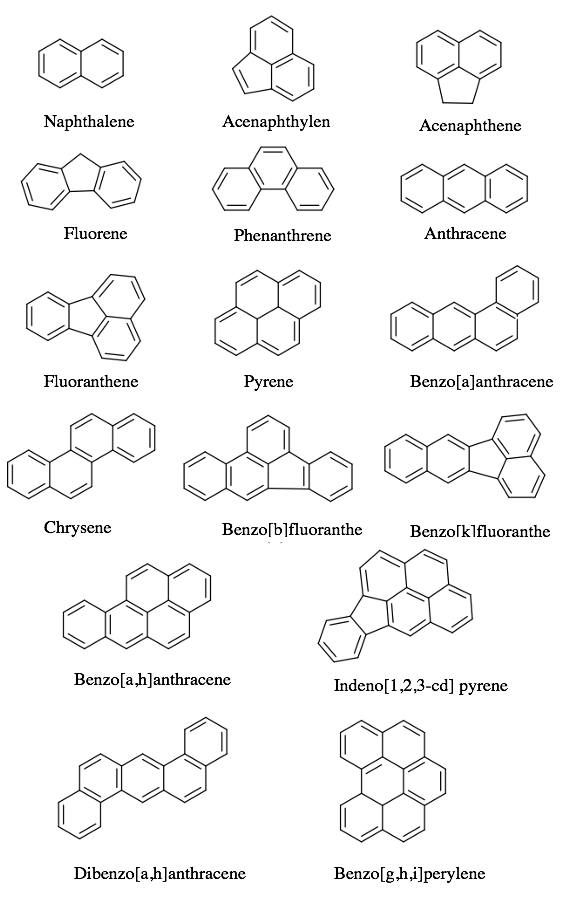


Figure S1 Structural Formula of 16 PAHs

**Figure S2SEM for biochar and ball-milling biochar: A, C-biochar; B, D-milling biochar**


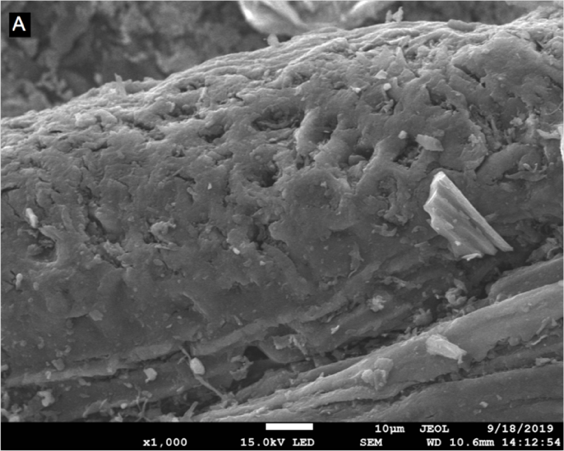

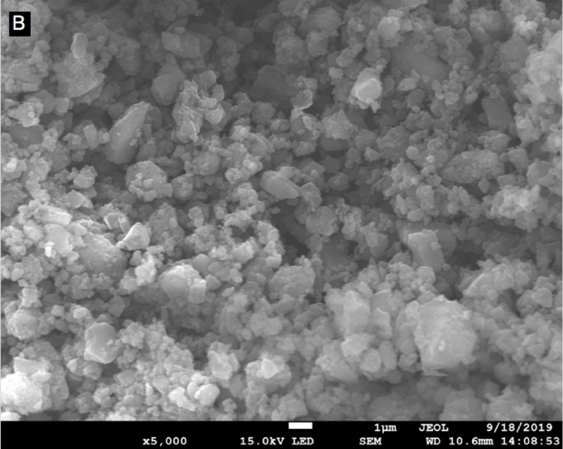


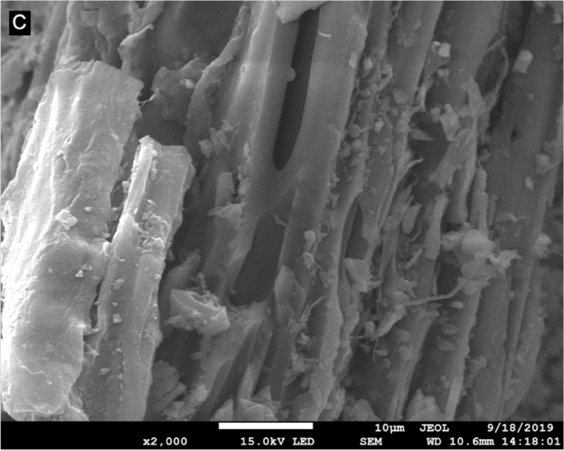

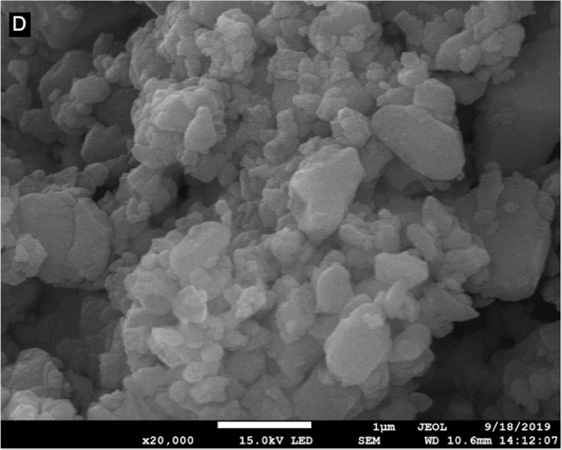


Figure S2 SEM for biochar and ball-milling biochar: A, C-biochar; B, D-milling biochar

**Figure S3 FT-IR spectra for biochar/ ball-milling biochar: BC-biochar, BM-ball-milled biochar**

Figure S3FT-IR spectra for biochar/ ball-milling biochar: BC-biochar, BM-ball-milled biochar.

**References**

Chen, J., Gu, B., LeBoeuf, E.J., Pan, H., Dai, S., 2002. Spectroscopic characterization of the structural and functional properties of natural organic matter fractions. Chemosphere 48, 59–68. https://doi.org/10.1016/S0045-6535(02)00041-3

Croué, J.-P., Benedetti, M.F., Violleau, D., Leenheer, J.A., 2003. Characterization and Copper Binding of Humic and Nonhumic Organic Matter Isolated from the South Platte River:  Evidence for the Presence of Nitrogenous Binding Site. Environ. Sci. Technol. 37, 328–336. https://doi.org/10.1021/es020676p

Dilling, J., Kaiser, K., 2002. Estimation of the hydrophobic fraction of dissolved organic matter in water samples using UV photometry. Water Research 36, 5037–5044. https://doi.org/10.1016/S0043-1354(02)00365-2

Hur, J., Schlautman, M.A., 2003. Using Selected Operational Descriptors to Examine the Heterogeneity within a Bulk Humic Substance. Environ. Sci. Technol. 37, 880–887. https://doi.org/10.1021/es0260824

Jaffrain, J., Gérard, F., Meyer, M., Ranger, J., 2007. Assessing the Quality of Dissolved Organic Matter in Forest Soils Using Ultraviolet Absorption Spectrophotometry. Soil Science Society of America Journal 71, 1851. https://doi.org/10.2136/sssaj2006.0202

Kalbitz, K., Geyer, S., Geyer, W., 2000. A comparative characterization of dissolved organic matter by means of original aqueous samples and isolated humic substances. Chemosphere 40, 1305–1312. https://doi.org/10.1016/S0045-6535(99)00238-6

Kalbitz, K., Schmerwitz, J., Schwesig, D., Matzner, E., 2003. Biodegradation of soil-derived dissolved organic matter as related to its properties. Geoderma 113, 273–291. https://doi.org/10.1016/S0016-7061(02)00365-8

Li, B., Wu, L., Xu, Y., Qin, Y., 2017. Relationship between functional groups of soil dissolved organic carbon and CO2 emissions with crop residues incorporation to soil 36, 2535–2543.

Li, D., He, X.S., Xi, B.D., Wei, Z.M., Pan, H.W., Cui, D.Y., 2014. Study on UV–Visible Spectra Characteristic of Dissolved Organic Matter during Municipal Solid Waste Composting. Advanced Materials Research 878, 840–849. https://doi.org/10.4028/www.scientific.net/AMR.878.840

Scott, M.J., Jones, M.N., Woof, C., Simon, B., Tipping, E., 2001. The molecular properties of humic substances isolated from a UK upland peat system A temporal investigation. Environment International 14.
